# Opportunities and challenges for time-resolved studies of protein structural dynamics at X-ray free-electron lasers

**DOI:** 10.1098/rstb.2013.0318

**Published:** 2014-07-17

**Authors:** Richard Neutze

**Affiliations:** Department of Chemistry and Molecular Biology, University of Gothenburg, PO Box 462, 40530 Gothenburg, Sweden

**Keywords:** X-ray free-electron lasers, structural biology, serial femtosecond crystallography, time-resolved diffraction, time-resolved wide angle X-ray scattering

## Abstract

X-ray free-electron lasers (XFELs) are revolutionary X-ray sources. Their time structure, providing X-ray pulses of a few tens of femtoseconds in duration; and their extreme peak brilliance, delivering approximately 10^12^ X-ray photons per pulse and facilitating sub-micrometre focusing, distinguish XFEL sources from synchrotron radiation. In this opinion piece, I argue that these properties of XFEL radiation will facilitate new discoveries in life science. I reason that time-resolved serial femtosecond crystallography and time-resolved wide angle X-ray scattering are promising areas of scientific investigation that will be advanced by XFEL capabilities, allowing new scientific questions to be addressed that are not accessible using established methods at storage ring facilities. These questions include visualizing ultrafast protein structural dynamics on the femtosecond to picosecond time-scale, as well as time-resolved diffraction studies of non-cyclic reactions. I argue that these emerging opportunities will stimulate a renaissance of interest in time-resolved structural biochemistry.

## 1. Introduction

Synchrotron radiation has had dramatic impact on the life sciences. The most visible application of synchrotron radiation is structural biology, which will soon pass the milestone of 100 000 structures deposited in the Protein Data Bank (www.pdb.org), of which approximately 90% are now solved using synchrotron radiation. Many major challenges in structural biology have yielded high-resolution X-ray structures, including large protein : RNA complexes [1], DNA complexes [2] and challenging membrane protein structures [3]. G-protein-coupled receptors (GPCRs), long believed to be intractable to crystallization and crystallography due to their inherent flexibility, have recently yielded to a combination of ingenious protein engineering [4,5] and cryo-microcrystallography at synchrotron-based experimental stations [6]. Other life science applications of synchrotron radiation include the development of X-ray microscopy and coherent X-ray imaging of biological samples such as unstained frozen cells [7,8]. Moreover, recent technical advances in single particle electron microscopy (EM) have led to spectacular progress with EM structures of frozen single particles reported to near atomic resolution [9,10]. Electron tomography of entire cells [11] is approaching the long-term dream of recovering three-dimensional images of cells to a resolution that enables individual proteins and macromolecular assemblies to be recognized.

X-ray free-electron lasers (XFELs) facilitate entirely different structural approaches by providing extremely short X-ray pulses with approximately 10^12^ X-ray photons/pulse that can be focused to a sub-micrometre focal spot [12]. This corresponds to a jump in peak X-ray brilliance of ten orders of magnitude over storage ring sources: *which is the difference between a casual walk and travelling at the speed of light* [13]. Thus, XFEL radiation represents an example of disruptive technology, whereby technical advances create fundamentally new opportunities for scientific research. Early life science applications of XFEL radiation [14] have focused upon the development of serial femtosecond crystallography (SFX) as a high-resolution structural method [15–21]; the application of time-resolved approaches to SFX [22,23]; and the development of coherent X-ray imaging of single viruses [24] and cells [25]. In this commentary, I discuss this recent progress and argue that XFEL radiation should be viewed as a complement to synchrotron radiation and single particle EM that will open up new scientific opportunities as well as accelerate the rate of progress in structural biology. A future challenge for XFEL sources is to enable discoveries in life science that could not be realized using synchrotron radiation or cryo-EM. I believe that one important avenue will be to probe the structural dynamics of biomolecules from atomic to cellular length scales on time-scales from femtoseconds to milliseconds.

## 2. Diffraction before destruction

Fourteen years ago, we combined molecular dynamics simulations with X-ray scattering calculations to argue that femtosecond X-ray pulses would facilitate a new regime with respect to the X-ray exposure that can be tolerated by biological samples [26]. By rapidly collecting X-ray scattering data from a sample undergoing an X-ray damage-induced Coulomb explosion, we reasoned that it would be possible to recover interpretable diffraction data if the X-ray exposure was shorter in duration than the time-scale needed for a biological sample to explode. The idea that extremely intense X-ray pulses could facilitate the recording of X-ray scattering images before a biological specimen is destroyed was previously mooted by Solem & Baldwin [27], but that discussion was not quantitative with respect to radiation damage processes and hypothesized that sub-nanosecond soft X-ray sources being developed in the 1980s would attain the necessary peak brilliance.

Further quantitative studies expanded and improved upon the physical model of the radiation damage process [28] and explored possible approaches for aligning and inverting X-ray scattering data from single particles [29–31]. The upshot of this body of theoretical work is that the major conclusions of our analysis [26] have stood the test of time: X-ray pulses of a few tens of femtoseconds or shorter will create a new opportunity for pushing back the traditional radiation damage limits of structural biology [32,33]; at the upper limits of the allowed X-ray fluence, it will be possible to collect interpretable X-ray scattering data from single large biological molecules such as viruses; and interpretable X-ray diffraction data will be recoverable from protein crystals only a few unit cells across. These considerations have featured among several early experiments at the Linac Coherent Light Source (LCLS) [14], the world's first hard XFEL [12], and experimental data have demonstrated that the diffraction power of microcrystals falls off significantly as the X-ray pulse duration is extended beyond 70 fs [34].

## 3. Serial femtosecond crystallography

One early life science application of XFEL radiation was our demonstration that it was possible to collect interpretable diffraction data from microcrystals of the large membrane protein complex photosystem I [15]. This achievement by Chapman *et al.* was founded upon several parallel technical advances including the first lasing at the LCLS [12]; the commissioning of the first LCLS beamline which operated at a wavelength of approximately 6 Å [35]; the construction of a versatile in-vacuum X-ray detector environment [36]; the development of microjet technologies for sample injection [37]; and the development of new software analysis tools for processing and merging serial crystallography data [38,39]. What these pioneering experiments showed was that it was possible to collect interpretable X-ray diffraction data using extreme intensity XFEL pulses from a series of measurements from independent crystals of micrometre to sub-micrometre size, even though each and every microcrystal exposed to the XFEL beam was vaporized. The idea of diffraction before destruction [26] was an experimental fact!

**Figure 1.**
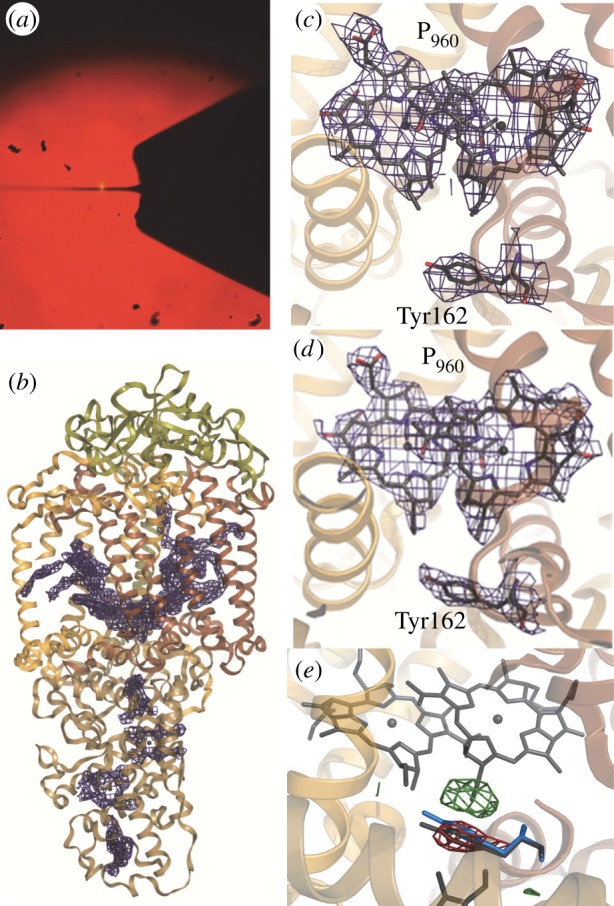
SFX and time-resolved Laue diffraction studies of the photosynthetic reaction centre of *Bl. viridis* (RC_vir_). (*a*) Injection of RC_vir_ microcrystals into an XFEL beam using the Spence microjet. (*b*) SFX structure of RC_vir_ solved to 3.5 Å resolution. (*c*) Close-up view of the SFX electron density map near the special pair (P_960_) and near TyrL162. (*d*) Similar view as in (*c*), but of a Laue diffraction electron density map to 2.95 Å resolution. All 2F^obs^–F^calc^ electron density maps (blue) are contoured at 1.0σ. (*e*) Difference density (green positive density; red negative density, contoured at 4.0σ) illustrating the structural changes induced by light (the movement of TyrL162 towards the special pair) captured using time-resolved Laue diffraction. These figures are reproduced with permission from [16] (*a*), [20] (*b*,*c*) and [42] (*d*,*e*, with modifications).

Johansson *et al.* [16] also demonstrated that membrane protein microcrystals could be grown in a lipidic sponge phase environment and injected directly into the focused LCLS beam. Unlike nano/microcrystals of photosystem I [15], the microcrystals of the *Blastochloris viridis* photosynthetic reaction centre were not isomorphous to their larger crystal phase form [40], packing in a new space-group and having one very long (398 Å) cell axis. When shorter wavelength X-rays became available at the coherent X-ray imaging (CXI) beamline [41] of the LCLS these microcrystals diffracted to 2.8 Å resolution and data were processed and the structure refined to 3.5 Å resolution [20]. Despite the relatively low multiplicity (approx. 27) in these studies, convincing electron density was recovered (figure 1*a,b*) and no evidence of X-ray-induced radiation damage were observed within the structure. Other membrane protein SFX structures include photosystem II to 5.7 Å resolution [23], and the human serotonin receptor, a GPCR [21], has yielded a crystal structure to 2.8 Å resolution (multiplicity of 1150), for which somewhat larger crystals were grown using a lipidic cubic phase (LCP) crystallization matrix [43]. Because of the extremely high medical importance of GPCRs [5], which form a major class of pharmaceutical targets, SFX studies of this family of membrane proteins are likely to become an important future application of XFEL radiation in biology.

SFX structures to high resolution have been recovered from microcrystals of the soluble protein cathepsin B [18] (2.1 Å resolution; 7808 multiplicity) grown *in vivo* within an insect cell expression host [44]. This is an extremely elegant approach to the problem of micro-crystallization, but it remains to be seen if *in vivo* crystallization can be developed into a widely used generic approach for challenging problems in structural biology. Proof of principle studies of microcrystals of the tried-and-trusted model system lysozyme both currently hold the resolution record for an SFX structure at 1.9 Å resolution [17] and have demonstrated the possibility of heavy-atom phasing of protein structures [19]. This demonstration of *de novo* phasing by Barends *et al*. using XFEL radiation is an important milestone since it lays the foundations for solving structures of macromolecules without known homology models, which was by no means obvious given the challenges of merging diffraction data from thousands of randomly oriented microcrystals of varying shape and size.

## 4. Potential impact of serial femtosecond crystallography

SFX holds promise for accelerating the rate of progress in challenging problems in structural biology since it creates new opportunities to extract structural information from thousands of microcrystals that are too small to yield complete data using synchrotron radiation. On the other hand, XFEL-based SFX will ultimately be judged against the extent to which new structural insights emerge that would not be accessible using synchrotron radiation or single particle cryo-EM. To this end, the first proof-of-principle studies at the LCLS using model systems of known structure have been important, but higher resolution X-ray structures of photosystem I [45], photosystem II [46], the photosynthetic reaction centre [40] and lysozyme [47] have all been solved using synchrotron radiation. Given the pace of developments, examples will surely soon emerge where diffraction data recorded at an XFEL is of higher quality than that attainable at a synchrotron source. Moreover, one key aspect is that diffraction data recorded from radiation sensitive proteins do not show signs of radiation damage [17,20] and this can be very important for some proteins such as photosystem II [23] for which X-ray-induced reduction of the manganese cluster has been a hot issue.

Another challenge with early SFX approaches is the volume of protein required for XFEL studies using the Spence microjet [37,48], which consumes approximately 1 ml of crystallization drops per hour. As a major challenge of difficult structural biology targets is producing purified protein for crystallization, it is not realistic to expect that hundreds of milligrams of crystals can routinely be made available for XFEL studies. This issue is being addressed through the development of new injection technologies, including the highly viscous LPC microjet [21,49] that uses approximately 1% of the volume of the liquid phase microjet.

SFX has already motivated a rethink of how diffraction data can be collected using dedicated microfocus protein crystallography beamlines at a storage ring. The advent of rapid readout X-ray detectors [50] and X-ray choppers [51] creates the possibility of recording serial crystallography data from thousands of microcrystals either delivered at room temperature in a slow moving LCP microjet [49], using other sample delivery technologies [52], or using multiple crystals frozen within cryo-loops. The need to merge crystallographic data from dozens of large crystals has been well known to crystallographers studying virus particles [53], which typically produce crystals with very large unit cell and are therefore very sensitive to mosaic spread when freezing. XFEL-based SFX has added a new dimension to this challenge by demonstrating that data from tens of thousands of microcrystals can be merged successfully. This idea should be adapted and pushed to its limits using synchrotron radiation [6], since the X-ray dose of 33 MGy used in high-resolution studies at the LCLS of the reaction centre [20], cathepsin B [18] and lysozyme [17] is consistent with the dose that can be delivered to frozen crystals using synchrotron radiation [33]. The potential advantages of synchrotron base serial crystallography will be the relative ease of access to storage ring facilities, user familiarity in transporting frozen crystals to an experiment and the maturity of support technologies. Synergies will emerge as scientists at both storage ring and XFEL sources collaborate with users to accelerate the pace of progress in life science, with micro-crystallization conditions being optimized at storage rings but the published data ultimately being collected at an XFEL; or microcrystal leads being identified first using XFEL radiation on unfrozen samples but these conditions being optimized to yield larger crystals suitable for cryo-data collection at a storage ring.

## 5. Time-resolved Laue diffraction and time-resolved serial femtosecond crystallography

XFEL-based time-resolved structural studies [54] create opportunities for discoveries in life science that are not accessible using synchrotron radiation. Since 1996, the push to record ultrafast time-resolved movies of protein structural changes using Laue diffraction [55–60] has become limited by the electron bunch duration of approximately 100 ps. XFEL radiation offers extremely brilliant X-ray pulses of approximately 40 fs, and thus opens up new possibilities for ultrafast time-resolved diffraction studies of biomolecules. The classical Laue diffraction approach, of collecting both dark reference and light-activated images for each and every oscillation from the same crystal, may be difficult to apply at XFELs because self-amplified stimulated emission (SASE) produces an X-ray spectrum that is stochastic, with considerable pulse-to-pulse variation. Spectral fluctuations are difficult when processing Laue diffraction data due to the need to normalize the measured scattering intensities against the X-ray fluence through the crystal. XFEL seeding [61] provides a potential solution to the problem of SASE but at the cost of a very narrow XFEL bandwidth, which is advantageous in most applications but not appropriate for time-resolved Laue diffraction. This is because a narrow bandwidth results in many observations being partials and demands that many different oscillation angles be sampled, making the experiment sensitive to laser pump and X-ray probe induced damage. It is therefore likely that recovering convincing time-resolved electron density changes on the ultrafast time-scale from a single large crystal will only be possible for exceptionally robust crystals, if at all.

We previously performed a time-resolved Laue diffraction study of large crystals of the *Bl. viridis* photosynthetic reaction centre [42] and observed a light-induced movement of TyrL-162 towards the special pair of bacteriochlorophylls P_960_ 3 ms after photoactivation (figure 1*e*). It is striking that the 2mF^obs^-DF^calc^ SFX electron density recovered from the same reaction centre by merging diffraction data recorded from 1175 microcrystals (figure 1*d*, [20]) is very similar to the quality of the 2mF^obs^-DF^calc^ Laue diffraction map recovered from merging data from three large crystals (figure 1*d*). This suggests that time-resolved SFX offers an alternative path to achieving high-resolution structural information on ultrafast protein dynamics at an XFEL. This optimism comes with the caveat that it remains to be demonstrated that the merging of partial reflections from microcrystals of different sizes, as well as the influence of pulse-to-pulse variations in the XFEL spectrum and intensity, will yield intensity estimates sufficiently accurate to measure small laser-induced structural changes to high resolution.

Three advantages are apparent for time-resolved SFX when compared with time-resolved Laue diffraction approaches using large crystals: (i) microcrystals are much smaller and therefore have lower optical density, which allows a more homogeneous excitation of molecules within microcrystals; (ii) since the sample is continuously replaced the experimental data are not sensitive to the accumulated X-ray- and pump laser-induced damage, and (iii) for the same reasons, the systems of study are not restricted to probing reactions that return to their resting state, potentially opening up the study of chemically driven enzymatic reactions at room temperature to time-resolved diffraction. Moreover, because SFX neatly avoids the presence of X-ray damage-induced artefacts in the electron density, this may prove telling for high-resolution studies of chemical reactions for which artefacts of X-ray damage have been controversial in cryo-trapping studies [62]. These points are critical for the field of time-resolved crystallography to expand its sphere of systems of study and become a more mainstream, integrated approach within structural biology.

The first attempts to apply time-resolved SFX to study reaction dynamics in microcrystals probed a complex of photosystem 1 and ferodoxin, which showed disordering of the microcrystals a few microseconds after photoactivation [22]; and time-resolved SFX studies of photosystem II, which revealed that the oxygen evolving cluster is not photo-reduced by X-rays and the S2 state cannot be distinguished from the S1 (resting) state at 6 Å resolution [63]. In my view, these pioneering efforts to develop time-resolved SFX at an XFEL lay promising ground for future time-resolved studies of a broader set of biological reactions than have been probed using time-resolved Laue diffraction to date.

## 6. Time-resolved wide angle X-ray scattering

Solution phase time-resolved wide angle X-ray scattering (WAXS) is another promising technique for observing structural changes in proteins. This method builds upon earlier studies of the reaction dynamics of small photochemical molecules [64–68] and was first extended to probe the ultrafast dynamics of proteins by Cammarata *et al*. [69], who recorded WAXS data following the photo-dissociation of carbon monoxide from tetrameric haemoglobin and cytochrome *c*. A series of later studies probed the reaction dynamics of other light-triggered reactions such as the photo-dissociation of CO from the haem groups of myoglobin [60,70–72] and homodimeric haemoglobin [69], as well as chromophore isomerization-driven reactions within photoactive yellow protein [73,74], bacteriorhodopsin [75] and proteorhodopsin [75–77].

**Figure 2.**
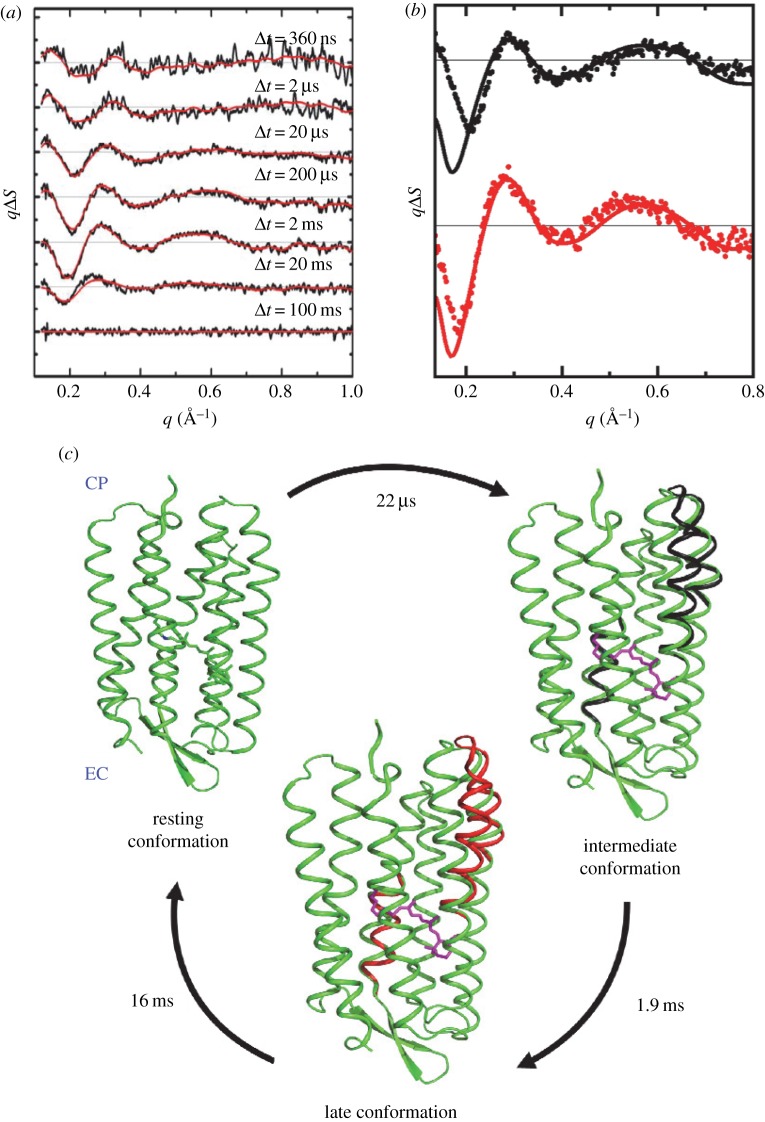
Time-resolved WAXS studies of light-driven structural changes in bacteriorhodopsin. (*a*) Time-resolved WAXS difference data (Δ*S*(*q*,Δ*t*)) as a function of the time-delay (Δ*t*) following photoactivation by a short visible laser pulse. (*b*) Two difference WAXS basis spectra extracted from spectral decomposition of the data shown in (*a*) (dots), and the theoretical fits to this data (solid lines), for an intermediate time-scale (black) and slower (red) component of the data. (*c*) Refined conformational changes in bacteriorhodopsin recovered by a best-fit analysis to the experimental difference WAXS basis spectra shown in (*b*). These figures are reproduced with permission from [75].

Figure 2*a* illustrates the time-resolved WAXS difference data recorded from bacteriorhodopsin from 360 ns to 100 ms after photo-excitation [75] after the effects of heating on the WAXS difference data were removed. Oscillations are observed in the difference WAXS data that correlate with changes within the protein structure. To fit the difference WAXS data (figure 2*b*), difference spectra were predicted using structural changes modelled from movements of α-helices that had previously been observed in low-temperature trapping [78] and mutation [79] studies of bacteriorhodopsin [80] (figure 2*c*).

The advantage of time-resolved WAXS is that it is a generic approach for which protein movements are not restricted by the packing of a crystal lattice and the quality of the scattering data that can be recovered is not compromised by these movements. The disadvantage is that the structural information is much less detailed than that recovered using diffraction methods, and while a number of structural refinement approaches have been explored [60,74,75,77,81], there are not yet any agreed standards for structural refinement against difference WAXS data. What should also be appreciated is that time-resolved WAXS observes only very small experimental difference in the X-ray scattering data (Δ*S*(*q*)/*S*(*q*) ∼ 0.1–1.0%) and hence the beamline and detector stability, and photon counting statistics, have all been limiting factors that have only been overcome due to constant technical advancements at dedicated storage ring time-resolved beamlines.

XFEL applications of time-resolved WAXS appear to be particularly promising. First and foremost, a completely new possibility arises to perform time-resolved WAXS studies with sub-picosecond resolution. It will be exciting to see whether these promising approaches can truly deliver the dream of observing ultrafast structural changes in light-sensitive proteins on the sub-picosecond time-scale. At this time-scale, energy absorbed by buried chromophores will be rapidly dissipated. It was hypothesized almost three decades ago that some of this energy is dissipated as a protein conformational change that propagates at the speed of sound out from an epicentre and was coined a protein quake [82] as a nanoscopic analogy to the propagation of energy during an earthquake. Whether or not global ultrafast structural changes play any functional role in light-sensitive proteins [82–86] is a matter of considerable debate, and time-resolved WAXS at an XFEL offers a completely new approach for addressing this question.

A related scientific issue that will be observable using ultrafast time-resolved WAXS at an XFEL is the question as to how fast functionally important protein structural changes can occur. For example in bacteriorhodopsin, a light-driven proton pump, low-temperature crystallography methods have established that retinal isomerization displaces a key water molecule early on in the photocycle [80,87]. It is therefore of considerable interest to measure the time-scale at which local water movements can induce global protein conformational changes, and these most rapid motions were not captured in our earlier work using synchrotron radiation [75] (figure 2). Owing to the remarkable X-ray fluence, XFEL-based time-resolved WAXS studies on the picosecond to nanosecond time-scales will have major signal-to-noise benefits over synchrotron-based studies, allowing better quality data to be recorded to characterize the most rapid protein motions. Moreover, it may be possible to adapt the microjet sample injection developed for SFX to time-resolved WAXS studies [37,48]. If successful, this will mean that X-ray and laser-induced damage does not accumulate within the sample since each sampled volume is exposed only once to the pump laser and X-ray probe. By alternating light-and-dark images during data collection, it should be possible to account for fluctuations in the XFEL spectrum and the microjet itself, although robust statistical methods will need to be adapted from synchrotron-based time-resolved WAXS applications to deal with these factors.

A fundamental and very exciting development of XFEL-based time-resolved WAXS is the potential application of angular correlations when using an ultrafast X-ray probe [88,89]. The idea is that because the XFEL pulses are so short, proteins appear to be frozen in time during the X-ray exposure and this means that angular correlations that are usually washed-out in storage ring-based SAXS or WAXS studies are present in the XFEL-based experimental data. To fully exploit these new opportunities, considerable effort will be needed to minimize experimental noise and drift in solution phase XFEL experiments. Nevertheless, since time-resolved WAXS data are collected on a two-dimensional detector, it only makes sense to explore if the structural information that can be extracted from a time-dependence difference signal can be enriched by searching for angular correlations. I believe that these benefits, along with continuous development of time-resolved WAXS at storage ring sources, will see the sphere of application of time-resolved WAXS expand to probe the structural dynamics of a more diverse sample of proteins on time-scales from femtoseconds to seconds.

## 7. Generic approaches to reaction triggering

Time-resolved diffraction studies of protein reaction dynamics in four dimensions have been hamstrung by a lack of generic approaches to reaction triggering. One of the most promising aspects of XFEL-based approaches to time-resolved serial crystallography is the possibility to probe the structural dynamics of proteins and other macromolecules in their crystalline form without the requirements that the reaction is cyclic and returns to its resting state. This shift is potentially transformative for the entire field and arises because diffraction data are collected from each and every microcrystal only once. Two key limitations of time-resolved Laue diffraction are the need (in practice, not in principle) to study photo-reversible reactions; and the high-sensitivity to disorder of the Laue method, meaning that the very structural change that you wish to observe may make the crystals unsuitable for Laue diffraction.

I foresee the potential for significant growth in possible approaches to reaction triggering using serial crystallography such as: rapid chemical mixing of reactants and microcrystals in microfluidic devices; slower mixing using caged compounds in combination with triggering the release of the reactant using a light or UV laser pulse; or slower mixing of reactants and microcrystals near 0°C and using short IR pulses used to heat the microcrystals before they interact with X-ray beam, thereby driving the reaction across a specific rate-limiting reaction barrier; the use of intense THz pulses to stimulate specific dynamic modes within proteins [90]; or the use of applied AC electric fields to drive resonances within molecules on the kHz to GHz frequencies [91]; or engineering light-sensitive triggers into proteins directly [92]. If several of these approaches prove successful then the opportunity arises for time-resolved crystallography and time-resolved WAXS to become considerably more mainstream structural techniques than they are today.

## 8. Conclusion

Synchrotron radiation has become a profoundly successful tool for structural biology. The first applications of synchrotron radiation in biology were motivated by the desire to record images of muscle contraction with millisecond time-resolution [93]. Although diffraction was demonstrated using synchrotron radiation in 1971 [94], no one at the time could have foreseen that, a generation later, thousands of X-ray structures of macromolecules would be solved annually using synchrotron radiation, or that powerful methods for phasing structures based upon anomalous diffraction [95,96] would emerge from the application of synchrotron radiation to structural biology. Similarly, single particle cryo-EM is a very beautiful technique that is rapidly advancing towards atomic resolution [9,10]. While some might argue that XFEL-based SFX [15] and coherent diffractive imaging [24] must compete with these mature structural methods, I believe that the challenge is to understand where storage ring data and cryo-EM studies can be complemented by the novel scientific opportunities created by XFEL radiation. At the end of the day, XFEL-based life science will be judged on the extent to which important new biological insights emerge that could not have been attained with other structural approaches.

In this opinion piece, I argue that one area of application where XFEL radiation has a key advantage is in time-resolved diffraction and WAXS studies of protein reaction dynamics, both because of the time-scale of the X-ray pulses accessing a completely new domain in structural biophysics and because of the extreme peak brilliance offering huge gains in terms of signal-to-noise over that which can be achieved using synchrotron radiation. XFEL radiation thus creates new opportunities to explore structural and functional hypotheses on atomic distances on time-scales from femtoseconds to seconds. In my view, this is an exciting time to participate in molecular biophysics because, as when life scientists first turned to synchrotron radiation [94,97], the potential for new discoveries using XFEL sources is immense. While it is likely that the most important future applications of XFEL radiation in biology are only now being imagined, I believe that protein structural dynamics and the functional influence of protein dynamics within the cell is one important sphere of life science on which these fourth generation X-ray sources will shed new light.
